# Reliability and Sensitivity of Cognitive Outcome Measures to Plasma *p* ‐tau217 in Mild Cognitive Impairment in the AI‐Mind study

**DOI:** 10.1002/alz70861_108841

**Published:** 2025-12-23

**Authors:** Alexander J Kaula, Nora K Stang, Nick Taptiklis, Christoffer Hatlestad‐Hall, Ira Haraldsen, Francesca K Cormack

**Affiliations:** ^1^ Cambridge Cognition, Cambridge UK; ^2^ Oslo University Hospital, Oslo Norway

## Abstract

**Background:**

Selecting cognitive endpoints which are both sensitive to Alzheimer's pathology and reliable over time is crucial for early‐stage intervention trials. The AI‐Mind study assesses a large Mild Cognitive Impairment (MCI) cohort longitudinally over two years, collecting physiological measures, traditional pencil‐and‐paper neuropsychology, and computerized CANTAB tasks. In this rich dataset, we examined sensitivity of both classic CANTAB measures and newly derived reaction‐time (RT) metrics from the Paired Associates Learning (PAL) task to plasma *p* ‐tau217. We also evaluated test‐retest reliability these measures across visits to identify optimal cognitive endpoints combining sensitivity and reliability.

**Method:**

Participants with MCI in the AI‐Mind study completed four assessments over two years. We analysed data from 540 participants with MCI. Participants completed five CANTAB tasks at baseline and up to visit four over two years (PAL, DMS, PRM, RVP, SWM) and three pencil and paper assessments at baseline (RAVLT, Category Fluency, Trail Making Test), alongside plasma *p* ‐tau217. Each cognitive measure from visit one was individually entered into an OLS regression predicting plasma *p* ‐tau217, controlling for demographic variables. Sensitivity was measured via absolute standardised beta coefficients. Reliability of CANTAB measures was assessed with ICC(3,1) (two‐way mixed‐effects, absolute‐agreement). Finally, we identified the intersection of measures ranking highest in sensitivity and reliability to highlight optimal cognitive endpoints.

**Result:**

Across all measures, significant (*p* < .05) associations with *p* ‐tau217 fell in a narrow band: classic CANTAB measures exhibited betas of .12‐.16, and new PAL RT features showed betas of .14‐.18. Among pencil and paper tests, RAVLT, TMT and Category Fluency metrics had betas of .10‐.16. In reliability analyses, CANTAB and PAL RT measures yielded ICCs between .50 and .60, reflecting moderate stability in this restricted‐range sample over 24 months. The intersection of the ten most sensitive and ten most reliable measures produced a set of seven measures.

**Conclusion:**

This univariate approach has highlighted a set of cognitive measures that balance sensitivity to plasma *p* ‐tau217 with adequate test‐retest reliability in MCI. These empirically selected endpoints offer practical candidates for outcome measures in future early‐phase clinical trials.

